# Challenges to testing COVID-19 in conflict zones: Yemen as an example

**DOI:** 10.7189/jogh.10.010375

**Published:** 2020-06

**Authors:** Ghulam N Dhabaan, Walid A Al-Soneidar, Nezar N Al-Hebshi

**Affiliations:** 1 Department of Microbiology, Mount Sinai Hospital & University Health Network – University of Toronto, Toronto, Canada; 2 Department of Epidemiology, Biostatistics, and Occupational Health, McGill University, Montreal, Canada; 3 Oral Microbiome Research Laboratory, Department of Oral Health Sciences, Kornberg School of Dentistry, Temple University, Philadelphia, Pennsylvania, USA

When news came in from China about a new coronavirus, no one anticipated the situation we are facing today: a pandemic paralyzing the global economy, billions of people staying at home, and health care systems on the verge of collapse even in developed countries. The latter has been particularly amplified by the lack of treatment protocols as well as shortages of ventilators and ICU beds. One can only imagine how devastating the pandemic would be in poor countries, especially those involved in armed conflicts, where the health care system faces additional challenges, including lack of adequate testing to identify infected cases. While this is applicable to many countries in the region, the scenario is worst in Yemen. The country has been fragmented by war, devastated by a five-year long blockade, and has not yet recovered from deadly cholera and diphtheria epidemics that started in 2016. The aim of this commentary is to highlight the unique challenges of COVID-19 testing in Yemen and propose potential solutions.

Before moving the spotlight to the Yemeni case, it is useful to provide an overview of the available COVID-19 testing technologies. As soon as the full genome sequences of SARS-COV-2 became available, several in-house assays based on reverse transcription-PCR (RT-PCR) were developed by leading health institutions [1]. Within months afterwards, hundreds of commercial diagnostic kits were registered with the Foundation for Innovative New Diagnostics (FIND) for validation by WHO partner laboratories [2], and several received emergency use authorization from the Food and Drug Administration (FDA) [3]. These kits fall into two main categories: molecular and immunological assays [4]. Molecular assays, predominantly RT-PCR, are based on detecting amplified virus-specific RNA sequences, which makes them highly sensitive. They are currently the gold-standard for COVID-19 testing. Immunological assays are further classified into two types: one works by identifying antibodies produced in response to infection (also called indirect or serological tests); the other is designed to detect viral proteins (direct or antigen detection tests). Since it can take up to 20 days for COVID-19 patients to make detectable antibodies [5], antibodies-assays are primarily useful for surveillance of the recovered rather than active cases diagnosis. They could help obtain estimates of mortality rates and make decisions about reopening the economy. On the other hand, antigen kits, just like RT-PCR, can be used for diagnosis of early infection. These kits can be a cost-effective alternative to RT-PCR, especially in resource limited settings such as countries with underdeveloped laboratories and health care systems as they can be used as point-of-care rapid tests [6,7]. The FDA has just authorized the first COVID-19 rapid antigen test [8].

Back to the Yemeni situation. Yemen has six central public health laboratories in the major cities of Sana’a, Aden, Mukalla, Taiz, Hodeida and Ibb. Four of these (those in Sana’a, Aden, Taiz and Mukalla) have the capacity of COVID-19 testing with RT-PCR under supervision of WHO. The latter has made reagents for 6700 RT-PCR tests available, and provided laboratory staff with technical training and standard of procedures [9]. This means Yemen is almost entirely dependent on a resource-limited setting supported by WHO which, in the best-case scenario, only allows testing a small number of highly suspected cases.

This is particularly problematic as PCR testing in conflict zones in likely to face many obstacles. RT-PCR is laborious, technique-sensitive and requires expertise for assay setup and interpretation of results. This is unlikely to be achieved given the short time and limited training provided by WHO. Due to frequent power outages, the stability and performance of the temperature-sensitive RT-PCR reagents could be compromised. Yemen also has a poor road network that has been largely destroyed by bombing, which makes the timely transporting of samples to reference laboratories extremely difficult.

While WHO with the local authorities continue to work on scaling up the RT-PCR testing capacity, testing coverage is not expected to significantly improve. It is time, therefore, to seriously consider introducing immunological tests as potential alternatives to RT-PCR to be used on a large scale. Immunological kits are cheap and do not require specialized training. Additionally, they work with common laboratory platforms (eg, ELISA) or can be used as point-of-care tests (eg, lateral flow assays) [4]. Antigen-detection kits are particularly important because they can be potentially used for detection of early infections [6,7]. It should be WHO’s priority to support companies with development and validation of rapid antigen tests, and ensure poor countries have early access to them. As mentioned above, the FDA has just granted EUA for the first COVID-19 rapid antigen test, which highlights the need for such kits not only in resource-limited settings but also in developed countries where they will alleviate the pressure on hospitals and specialized laboratories.

**Figure:**
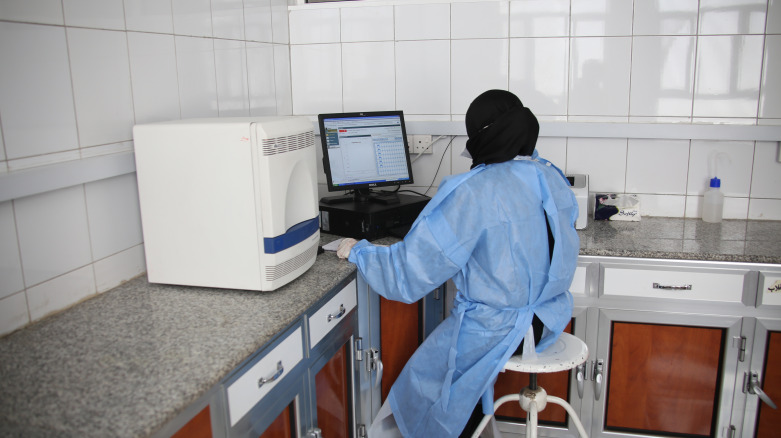
Photo: A PCR facility at the Central Public Health Laboratory in Taiz, Yemen, operating under limited resources and scarcity of tests. Photo taken by Taha Saleh (used with permission).

We recognize that antigen-detection kits may have suboptimal sensitivity, especially in samples with low viral load [6]; however, their diagnostic ability can be supplemented with antibodies tests, as antibodies can still be detectable in up to 50% of the patients within the first 7 days [6]. Serology tests can also be used for targeted screening of health care providers to identify those who developed immunity and can thus serve in the frontlines. However, they have to be selected with extreme care: in a very recent comparative study, only 3 out of 14 kits investigated were found to provide reliable results [5].

Combining antigen detection and serological test can allow the best of the two worlds – enhanced diagnostic ability of new cases and ease of use in resource-limited settings.
